# Complexity of Simple, Switched and Skipped Chaotic Maps in Finite Precision

**DOI:** 10.3390/e20020135

**Published:** 2018-02-20

**Authors:** Maximiliano Antonelli, Luciana De Micco, Hilda Larrondo, Osvaldo Anibal Rosso

**Affiliations:** 1 Facultad de Ingeniería, Universidad Nacional de Mar del Plata (UNMdP), Mar del Plata B7608FDQ, Argentina; 2 Instituto de Investigaciones Científicas y Tecnológicas en Electrónica (ICyTE), Universidad Nacional de Mar del Plata (UNMdP), Mar del Plata B7608FDQ, Argentina; 3 Consejo Nacional de Investigaciones Científicas y Técnicas (CONICET), Rivadavia 1917, Buenos Aires, Argentina; 4 Departamento de Informática en Salud, Hospital Italiano de Buenos Aires, Ciudad Autónoma de Buenos Aires C1199ABB, Argentina; 5 Instituto de Física, Universidade Federal de Alagoas (UFAL), Maceió 57072-900, Brazil; 6 Facultad de Ingeniería y Ciencias Aplicadas, Universidad de Los Andes, Monseñor Álvaro del Portillo 12455, Santiago, Chile

**Keywords:** chaos, finite precision, hardware implementaion, switching maps

## Abstract

In this paper we investigate the degradation of the statistic properties of chaotic maps as consequence of their implementation in a digital media such as Digital Signal Processors (DSP), Field Programmable Gate Arrays (FPGA) or Application-Specific Integrated Circuits (ASIC). In these systems, binary floating- and fixed-point are the numerical representations available. Fixed-point representation is preferred over floating-point when speed, low power and/or small circuit area are necessary. Then, in this paper we compare the degradation of fixed-point binary precision version of chaotic maps with the one obtained by using floating point 754-IEEE standard, to evaluate the feasibility of their FPGA implementation. The specific period that every fixed-point precision produces was investigated in previous reports. Statistical characteristics are also relevant, it has been recently shown that it is convenient to describe the statistical characteristic using both, causal and non-causal quantifiers. In this paper we complement the period analysis by characterizing the behavior of these maps from an statistical point of view using cuantifiers from information theory. Here, rather than reproducing an exact replica of the real system, the aim is to meet certain conditions related to the statistics of systems.

## 1. Introduction

In the last years digital systems became the standard in all experimental sciences. By using new programmable electronic devices such as DSP’s, ASIC’s or FPGA’s, experimenters are allowed to design and modify their own signal generators, measuring systems, simulation models, etc.

Nowdays, the chaotic systems are widely used in digital electronics in fields such as electromagnetic compatibility, encrypted secure communications, controlled noise generators, etc. [1,2,3,4,5]. These systems are specially interesting due to their extreme sensibility to initial conditions, nevertheless this characteristic is the source of the main difficulties at the time to implement them. In these fields the motivation of using chaotic systems is to genetrate sequences that meet certain requirements, rather than reproducing an exact replica of the real systems.

When a chaotic system is implemented in computers or any digital device, the chaotic attractor become periodic by the effect of finite precision, then only pseudo chaotic attractors can be generated [6,7]. Discretization may even destroy the pseudo chaotic behavior and consequently is a non trivial process [3,8].

In these new devices, floating- and fixed-point are the most common arithmetics. Floating-point is the more accurately solution but is not always recommended when speed, low power and/or small circuit area are required. A fixed-point solution is better in these cases, nevertheless the feasibility of their implementation needs to be investigated.

The effect of numerical discretization over a chaotic map was recently addressed in [3,9,10,11]. In our previous work [3] we have explored the statistical degradation of the phases’ space for a family of 2D quadratic maps. These maps present a multiatractor dynamic that makes them very attractive as random number generator in fields like criptography, encoding, etc. Nepomuceno et al. [9] reported the existence of more than one pseudo-orbit of continuous chaotic systems when it is discretizated using different schemes. In [10] and [11], authors have proposed to use the value of the entropy to choose the number of bits in the fractional part, when maps are implemented in integer arithmetic.

Grebogi and coworkers [12] saw that the period *T* scales with roundoff ϵ as $T∼\epsilon^{-d/2}$ where *d* is the correlation dimension of the chaotic attractor. This issue was also addressed in [13], in this paper authors explore the evolution on the period *T* as consequence of roundoff induced by 2-based numerical representation. To have a large period *T* is an important property of chaotic maps, in [14] Nagaraj et al. studied the effect of switching over the average period lengths of chaotic maps in finite precision. They saw that the period *T* of the compound map obtained by switching between two chaotic maps is higher than the period of each map. Liu et al. [15] studied different switching rules applied to linear systems to generate chaos. Switching issue was also addressed in [16], the author considered some mathematical, physical and engineering aspects related to singular, mainly switching systems. Switching systems naturally arise in power electronics and many other areas in digital electronics. They have also interest in transport problems in deterministic ratchets [17] and it is known that synchronization of the switching procedure affects the output of the controlled system. Chiou and Chen [18] published an analysis of the stabilization and switching laws to design switched discrete-time systems. Recently, Borah et al. [19] presented a family of new chaotic systems and a switching based synchronization strategy.

Stochasticity and mixing are relevant to characterize a chaotic behavior. To investigate these properties several quantifiers were studied [20]. Entropy and complexity from information theory were applied to give a measure for causal and non causal entropy and causal complexity.

A fundamental issue is the criterium to select the probability distribution function (PDF) assigned to the time series, causal and non causal options are possible. Here we consider the non causal traditional PDF obtained by normalizing the histogram of the time series. Its statistical quantifier is the normalized entropy $H_{hist}$ that is a measure of equiprobability among all allowed values. We also considered a causal PDF that is obtained by assigning ordering patterns to segments of trajectory of length *D*. This PDF was first proposed by Bandt & Pompe in a seminal paper [21], the corresponding entropy $H_{BP}$ was also proposed as a quantifier by Bandt & Pompe. In [22] authors applied the causal complexity $C_{BP}$ to detect chaos. Among them the use of an entropy-complexity representation ($H_{hist}\times C_{BP}$ plane) and causal-non causal entropy ($H_{BP}\times H_{hist}$ plane) deserves special consideration [20,22,23,24,25,26].

Recently, amplitude information was introduced in [27] to add some immunity to weak noise in a causal PDF. The new scheme better tracks abrupt changes in the signal and assigns less complexity to segments that exhibit regularity or are subject to noise effects. Then, we define the causal entropy with amplitude contributions $H_{BPW}$ and the causal complexity with amplitude contributions $C_{BPW}$. Also, we introduce the modified planes $H_{hist}\times C_{BP}$ and $H_{BP}\times H_{hist}$.

Amigó and coworkers proposed the number of forbidden patterns as a quantifier of chaos [28]. Essentially they reported the presence of forbidden patterns as an indicator of chaos. Recently it was shown that the name forbidden patterns is not convenient and it was replaced by missing patterns (MP) [29], in this work authors showed that there are chaotic systems that present MP from a certain minimum length of patterns. Our main interest on MP is because it gives an upper bound for causal quantifiers.

Following [14], in this paper we study the statistical characteristics of five maps, two well known maps: (1) the tent (TENT) and (2) logistic (LOG) maps, and three additional maps generated from them: (3) SWITCH, generated by switching between TENT and LOG; (4) EVEN, generated by skipping all the elements in odd positions of SWITCH time series and (5) ODD, generated by discarding all the elements in even positions of SWITCH time series. Binary floating- and fixed-point numbers are used, these specific numerical systems may be implemented in modern programmable logic devices.

The main contribution of this paper is the study of how different statistical quantifiers detect the evolution of stochasticity and mixing of the chaotic maps according as the numerical precision varies. To illustrate this sequences generated by well known maps were used, and also sequences obtained by randomization methods like skipping and switching.

Organization of the paper is as follows: Section 2 describes the statistical quantifiers used in the paper and the relationship between their value and the characteristics of the causal and non causal PDF’s considered; Section 3 shows and discusses the results obtained for all the numerical representations. Finally Section 4 deals with final remarks and future work.

## 2. Information Theory Quantifiers

Systems that evolve over time are called dynamical systems. Generally, only measurements of scalar time series X(t) are accessible, these time series may be function of variables $V=\{v_{1},v_{2},\cdots ,v_{k}\}$ describing the underlying dynamics (i.e., dV/dt=f(V)). The goal is to infer properties and reach conclusions of an unknown system from the analysis of measured record of observational data. The key question would be to know how much information about the dynamics of the underlying processes can be revealed by the analysed data. A probability distribution function (PDF) *P* is typically used to quantify the apportionment of a time series X(t). Information Theory quantifiers can be defined as measures that characterize properties of the PDF associated with these time series, they allow to extract information about the studied system. We can see these quantifiers as metrics in the PDFs space, they enable to compare and classify different sets of data according to their PDFs where stochastic and deterministic are the two extremes of processes.

Here, we are concerned in chaotic dynamics, which are fundamentally *causal* and *statistical* in nature. For this reason, we are forced to use a different approach since a purely statistical approach ignores the correlations between successive values from the time series; and a causal approach focuses on the PDFs of data sequences.

The selected quantifiers are based on symbolic counting and ordinal pattern statistics. They make it possible to distinguish between the two main features: the *information content* of data and their *complexity*. It is important to highlight that rather than referring to a physical space, here we talk about a space of probability density functions.

In this section, we will introduce Information Theory quantifiers defined over discrete PDFs since discrete time series is our scope. Nevertheless, in [30] definitions for the continuous case can be found.

### 2.1. Shannon Entropy and Statistical Complexity

Entropy is a measure of the uncertainty associated with a physical process that is described by *P*. The Shannon entropy is considered as the most natural one [30] when dealing with information content.

Let define a discrete probability distribution $P=\{p_{i};i=1,\ldots ,N\}$ with $\sum_{i=1}^{N}p_{i}=1$, were *N* is the number of possible states of the system under study. Then, Shannon’s logarithmic information measure:

(1) $$S\left[P\right]\;=\;-\sum_{i=1}^{N}p_{i}\ln\left[p_{i}\right]\;$$

When $S\left[P\right]=S_{\min}=0$, the prediction of which outcome *i* will occur is a complete certainty. In this case, it is maximal the knowledge of the underlying process described by the probability distribution. On the contrary, we have minimal knowledge in a case of a uniform distribution $P_{e}=\left\{p_{i}=1/N;i=1,\ldots ,N\right\}$ since every outcome has the same probability of occurrence, and the uncertainty is maximal, i.e., $S\left[P_{e}\right]=S_{\max}=\ln N$. These two situations are extreme cases, therefore we focus on the ‘normalized’ Shannon entropy, 0≤H≤1, given as

(2) $$H\left[P\right]=S\left[P\right]/S_{\max}\;$$

On the other hand, there is not a unique definition of the concept of complexity. In this paper, the aim is to describe the complexity of the time series rather than that of the underlying systems. As stated by Kantz [31], complex data might be generated by “simple” systems, while “complicated” models can generate low complexity outputs. A quantitative complexity can be defined as a measure that assigns low values both to uncorrelated random data (maximal Shannon entropy) and also to perfectly ordered data (null Shannon entropy). Then, if we have an ordered sequence, such as a simple oscillation or trend, the statistical complexity would be low, and the same would happen with an unordered sequence, such as uncorrelated white noise. In the middle of this situation the characterization of data is more difficult hence the complexity would be higher.

We look forward a quantifier functional of a PDF, C[P], in order to measure the degree of complexity or of correlational structures. As depicted in [32] the functional is relate to organization, correlational structure, memory, regularity, symmetry, patterns, and other properties.

Rosso and coworkers [33] introduced a suitable *Statistical Complexity Measure* (SCM) *C*, that is based on the seminal notion advanced by López-Ruiz et al. [34]. SCM is defined as the product of a measure of information and a measure of disequilibrium and it is able to detect the distance from the equiprobable distribution of the accessible states.

This statistical complexity measure [33,35] is defined as follows:

(3) $$C\left[P\right]=Q_{J}\left[P,P_{e}\right]\cdot H\left[P\right]$$

*H* is the normalized Shannon entropy, see Equation 2, and $Q_{J}$ is the disequilibrium defined in terms of the Jensen–Shannon divergence $J[P,P_{e}]$, [36]. That is,

(4) $$Q_{J}\left[P,P_{e}\right]=Q_{0}J\left[P,P_{e}\right]=Q_{0}\left\{S\left[\left(P+P_{e}\right)/2\right]-S\left[P\right]/2-S\left[P_{e}\right]/2\right\}$$

$Q_{0}$ is a normalization constant such that $0\leq Q_{J}\leq 1$:

(5) $$Q_{0}\;=\;-2{\left\{\frac{N+1}{N}\ln\left(N+1\right)-\ln\left(2N\right)+\ln N\right\}}^{-1}\;$$

This value is obtained in a totally deterministic situation, where only one components of *P* has a no null value equal to one.

Note that the above introduced SCM depends on two different probability distributions: one associated with the system under analysis, *P*, and the other with the uniform distribution, $P_{e}$. Furthermore, it was shown that for a given value of *H*, the range of possible *C* values varies between a minimum $C_{min}$ and a maximum $C_{max}$, restricting the possible values of the SCM [37]. Thus, it is clear that important additional information related to the correlational structure between the components of the physical system is provided by evaluating the statistical complexity measure.

### 2.2. Determination of a Probability Distribution

The evaluation derived from the Information Theory quantifiers suppose some prior knowledge about the system; specifically for those previously introduced (Shannon entropy and statistical complexity), a probability distribution associated to the time series under analysis should be provided before. The determination of the most adequate PDF is a fundamental problem because the PDF *P* and the sample space Ω are inextricably linked. Usual methodologies assign to each value of the series X(t) (or non-overlapped set of consecutive values) a symbol from a finite alphabet $A=\{a_{1},\cdots ,a_{M}\}$, thus creating a *symbolic sequence* that can be regarded to as a *non causal coarse grained* description of the time series under consideration. In this case, information on the dynamics of the system is lost, such as order relations and the time scales.

If we want to incorporate *Causal information* we have to add information about the past dynamics of the system, this is done by the assignment of symbols of alphabet *A* to a portion of the trajectory. The Bandt and Pompe (BP) [21] method is a simple and robust methodology that compares neighboring values in a time series and, thus solves this issue. The causality property of the PDF allows quantifiers (based on this PDFs) to discriminate between deterministic and stochastic systems [38]. The steps are, first it is needed to create symbolic data by ranking the values of the series; and second to define by reordering the embedded data in ascending order. This last step is the same to make a phase space reconstruction, using embedding dimension *D* (pattern length) and time lag τ. The goal of quantifying the diversity of the ordering symbols is achieved. There is no need to perform model-based assumptions, because the appropriate symbol sequence arises naturally from the time series.

The procedure is the following:

- Given a series $\left\{x_{t};t=0,\Delta t,\cdots ,N\Delta t\right\}$, a sequence of vectors of length *D* is generated.
  (6) $$\left(s\right)⟼\left(x_{t-(d-1)\Delta t},x_{t-(d-2)\Delta t},\cdots ,x_{t-\Delta t},x_{t}\right)$$
  Each vector turns out to be the “history” of the value $x_{t}$. Clearly, the longer the length of the vectors *D*, the more information about the history would the vectors have but a higher value of *N* is required to have an adequate statistics.
- The permutations $\pi =(r_{0},r_{1},\cdots ,r_{D-1})$ of (0,1,⋯,D−1) are called “order of patterns” of time *t*, defined by:
  (7) $$x_{t-r_{D-1}\Delta t}\leq x_{t-r_{D-2}\Delta t}\leq \cdots \leq x_{t-r_{1}\Delta t}\leq x_{t-r_{0}\Delta t}$$
  In order to obtain an unique result it is considered $r_{i}<r_{i-1}$ if $x_{t-r_{i}\Delta t}=x_{t-r_{i-1}\Delta t}$. In this way, all the D! possible permutations π of order *D*, and the PDF P={p(π)} is defined as:
  (8) $$p\left(\pi \right)=\frac{♯\{s|s\leq N-D+1;(s)\;\mathrm{has}\mathrm{type}\;\pi \}}{N-D+1}$$
  In the last expression the ♯ symbol denotes cardinality.

From the time series an ordinal pattern probability distribution $P=\{p\left(\pi_{i}\right),i=1,\cdots ,D!\}$ is extracted. For this, a unique symbol π is obtained from the vector defined by Equation (8). For uniqueness, $r_{i}<r_{i-1}$ if $x_{s-r_{i}}=x_{s-r_{i-1}}$ is defined. With the following stationary assumption: for k≤D, the probability for $x_{t}<x_{t+k}$ should not depend on *t* need to be met for the applicability of the BP method. Regarding the selection of the parameters, Bandt and Pompe suggested working with 3≤D≤6 for typical time series lengths, and specifically considered a time lag τ=1 in their cornerstone paper.

Recently, the permutation entropy was extended to incorporate amplitude information. In order to avoid the loss of amplitude information, that would be a disadvantage of ordinal pattern statistics, weights are introduced to obtain a “weighted permutation entropy (WPE)” [27]. Non-normalized weights are computed for each temporal window for the time series *X*, such that

(9) $$w_{j}\;=\;\frac{1}{D}\sum_{k=1}^{D}{\left(x_{j+k-1}-{\bar{X}}_{j}^{D}\right)}^{2}$$

In the equation above ${\bar{X}}_{j}^{D}$ denotes the arithmetic mean of the current embedding vector of length *D* and its variance $w_{j}$ is then used to weight the relative frequencies of each ordinal pattern $p_{j}$. Originally, this technique was proposed to discriminate patterns immersed in low noise. We take advantage of the fact that the fixed points are not computed in the WPE.

We calculated the normalized permutation Shannon entropy *H* and the statistical complexity *C* from these PDFs, and the obtained values are denoted as:

- $H_{hist}$, is the normalized Shannon entropy applied to non-causal PDF $P_{hist}$;
- $H_{BP}$, is the normalized Shannon entropy applied to causal PDF $P_{BP}$;
- $H_{BPW}$, is the normalized Shannon entropy applied to causal PDF with amplitude contribution $P_{BPW}$;
- $C_{BP}$, is the normalized statistical complexity applied to causal PDF $P_{BP}$;
- $C_{BPW}$, is the normalized statistical complexity applied to causal PDF with amplitude contribution $P_{BPW}$.

### 2.3. Information Planes

A particularly useful visualization of the quantifiers from Information Theory is their juxtaposition in two-dimensional graphs. These diagnostic tools were shown to be particularly efficient to distinguish between the deterministic chaotic and stochastic nature of a time series since the permutation quantifiers have distinct behaviors for different types of processes. Four information planes are defined:

1. Causal entropy vs. non-causal entropy, $H_{BP}\times H_{hist}$;
2. Causal entropy with amplitude contributions vs. non-causal entropy, $H_{BPW}\times H_{hist}$;
3. Causal causal complexity vs. causal entropy, $C_{BP}\times H_{BP}$;
4. Causal causal complexity with amplitude contributions vs. causal entropy with amplitude contributions, $C_{BPW}\times H_{BPW}$.

**Figure 1:**
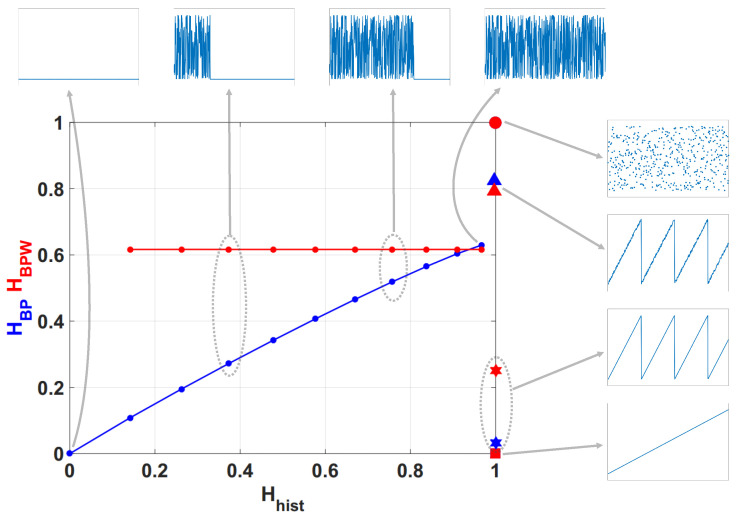
Causal-Non causal Entropy plane, values of the quantifiers for some characteristic signals, from purely deterministic through purely random (see text).

In Figure 1 we show the planes $H_{BP}\times H_{hist}$ and $H_{BPW}\times H_{hist}$ overlapped. In the resulting plane a higher value in any of the entropies, $H_{BP}$, $H_{BPW}$ or $H_{hist}$, implies a more uniformity in the involved PDF. The point (1,1) represents the ideal case with uniform histogram and uniform distribution of ordering patterns. We show some relevant points as example. Ideal white random sequences with uniform distribution gives a point at $\left(H_{hist},H_{BP}\right)=\left(1,1\right)$ represented by a blue circle, a red circle in the same position shows the results when amplitude contributions are included $\left(H_{hist},H_{BPW}\right)=\left(1,1\right)$. If we sort the vector generated by ideal white random generator in an ascendant way, resulting points are shown by a blue square $\left(H_{hist},H_{BP}\right)=\left(1,0\right)$ and a red square $\left(H_{hist},H_{BPW}\right)=\left(1,0\right)$, in both cases the squares are overlapped at point (1,0) this example illustrates the complementarity of the information provided by $H_{hist}$ and $H_{BP}$.

Blue and red stars show $\left(H_{hist},H_{BP}\right)$ and $\left(H_{hist},H_{BPW}\right)$ respectively, applied to a sawtooth signal. Values are perfectly distributed in all interval but only a few ordering patterns appear, this explains the high $H_{hist}$ and low $H_{BP}$. The frequency of occurrence of low amplitude patterns is higher than the one of high amplitude patterns, then the PDF with amplitude contributions is more uniform and $H_{BPW}$ is a little higher than $H_{BP}$. When sawtooth signal is contaminated with white noise $H_{BP}$ and $H_{BPW}$ are increased as shown with blue and red triangles. Clearly, new ordering patterns appear and both $H_{BP}$ and $H_{BPW}$ show higher values than non-contaminated case. However the growth of $H_{BPW}$ is smaller than $H_{BP}$ showing that the technique of recording amplitude contributions adds some immunity to noise.

Finally, we evaluated the quantifiers for a sequence of a logistic map that converges to a fixed point, in all cases the length of data vector remains constant and the length of transitory is variable. Results obtained without amplitude contributions are depicted in blue dots, they converge to $\left(H_{hist},H_{BP}\right)=\left(0,0\right)$ as the length of transitory is shorter, however $H_{BPW}$ (in red points) remains constant for all the cases. The last point in $\left(H_{hist},H_{BP}\right)=\left(0,0\right)$ corresponds to a vector of zeros, in this case the histogram of ordering patterns with amplitude contributions is also a zero vector and $H_{BPW}$ can not be calculated. Through this last example, we show that the convergence to a fixed point can be detected by the join information of $H_{BP}$ and $H_{BPW}$.

**Figure 2:**
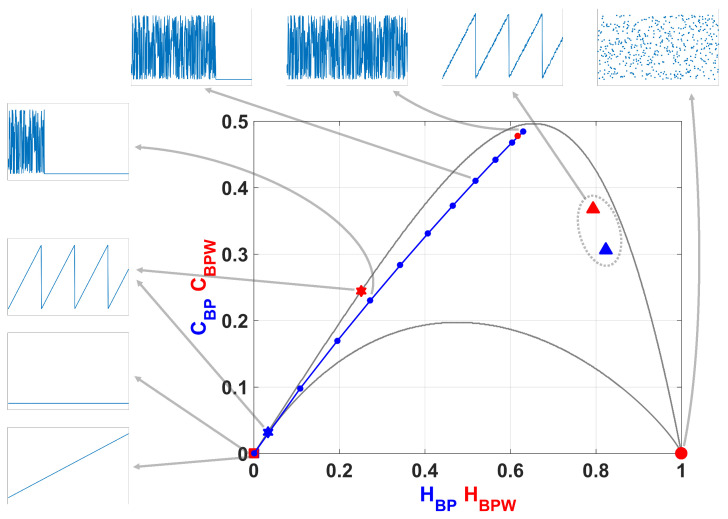
Causal Entropy-Complexity plane, values of the quantifiers for some characteristic signals, from purely deterministic trougth purely random (see text).

In Figure 2 we show the causality plane $H_{BP}\times C_{BP}$ with and without amplitude contributions. The same sample points are showed to illustrate the planar positions for different data vectors. We can see that not the entire region $0<H_{BP}<1$, $0<C_{BP}<1$ is achievable, in fact, for any PDF the pairs (H,C) of possible values fall between two extreme curves in the plane $H_{BP}\times C_{BP}$ [39].

Chaotic maps present high values of complexity $C_{BP}$ (close to the upper complexity limit),and intermediate entropy $H_{BP}$ [22,40]. In the case of regular processes the values of entropy and complexity are close to zero. Stochastic processes that are uncorrelated present $H_{BP}$ near one and $C_{BP}$ near zero. Ideal random systems having uniform Bandt & Pompe PDF, are represented by the point (1,0) [41] and a delta-like PDF corresponds to the point (0,0). In both information planes $H_{BP}\times H_{hist}$ in Figure 1 and $H_{BP}\times C_{BP}$ in Figure 2, stochastic, chaotic and deterministic data are clearly localized at different planar positions.

We also used the number of missing patterns MP as a quantifier [29]. As shown recently by Amigó et al. [42,43,44,45], in the case of deterministic maps, not all the possible ordinal patterns can be effectively materialized into orbits. Thus, for a fixed pattern-length (embedding dimension *D*) the number of missing patterns of a time series (unobserved patterns) is independent of the series length *N*. Remark that this independence does not characterize other properties of the series such as proximity and correlation, which die out with time [43,45]. In this paper, we use the MP to calculate an upper bound to the $H_{BP}$ and $H_{BPW}$.

A complete description and discussion about these quantifiers is out of scope of this manuscript. However, it can be found in [23,25,29,34,37,46,47,48].

## 3. Results

Five pseudo-chaotic maps were studied, two simple maps and three combination of them. For each one we have used numbers represented by floating-point (double presicion 754-IEEE standard) and fixed-point numbers with 1≤B≤53, where *B* is the number of bits that represents the fractional part. Time series were generated using 100 randomly chosen initial conditions within their attraction domain (interval [0,1]), for each one of these 54 number precisions. The studied maps are logistic (LOG), tent (TENT), sequential switching between TENT and LOG (SWITCH) and skipping discarding the values in the odd positions (EVEN) or the values in the even positions (ODD) respectively. In the following, all the results will be obtained from the pseudo-chaotic version of the LOG, TENT, SWITCH, EVEN and ODD maps.

Logistic map is interesting because it is representative of the very large family of quadratic maps. Its expression is:

(10) $$x_{n+1}=4\;x_{n}\;\left(1-x_{n}\right)$$

with $x_{n}\in R$.

Note that to effectively work in a given representation it is necessary to change the expression of the map in order to make all the operations in the chosen representation numbers. For example, in the case of LOG the expression in binary fixed-point numbers is:

(11) $$x_{n+1}=4\;\epsilon \;\mathrm{floor}\left\{\frac{x_{n}\left(1-x_{n}\right)}{\epsilon }\right\}$$

with $\epsilon =2^{-B}$ where *B* is the number of bits that represents the fractional part.

This rounding technique is the same as that used in [4,12,14] and has some advantages, such as, it is algorithmically easy to implement and is independent of the platform where it is used, as long as *B* is lower than the mantissa of the arithmetic of the local machine. In our case, the results were obtained with an Intel I7 PC, which has an ALU with IEEE-754 floating point standard with double precision, which limits the method to B≤53 bits.

Floating point representation does not constitute a field, wherein the basic maths properties, such as distributive, associative, are preserved. However, in fixed point arithmetics the exponent does not shift the point position and some properties as distributive remains. Also, we adopt the floor rounding mode because it arises naturally in a FPGA implementation.

The TENT map has been extensively studied in the literature because theoretically it has nice statistical properties that can be analytically obtained. For example it is easy to proof that it has a uniform histogram and consequently an ideal $H_{hist}=1$. The Perron-Frobenius operator and its corresponding eigenvalues and eigenfunctions may be analytically obtained for this map [49].

Tent map is represented with the equation:

(12) $$x_{n+1}=\left\{\begin{array}{cc} u\;x_{n} & ,\;\mathrm{if}\;0\leq x_{n}\leq 1/u\\ \frac{u}{1-u}\;\left(1-x_{n}\right) & ,\;\mathrm{if}\;1/u<x_{n}\leq 1 \end{array}\right.$$

with $x_{n}\;and$ u ∈R.

In base-2 fractional numbers rounding, Equation (12) becomes:

(13) $$x_{n+1}=\left\{\begin{array}{cc} \epsilon \;\mathrm{floor}\{\frac{1}{\epsilon }\mu \;\left(x_{n}\right)\} & ,\;\mathrm{if}\;0\leq x_{n}\leq \mu^{-}\\ \epsilon \;\mathrm{floor}\{\frac{1}{\epsilon }\rho \;\left(1-x_{n}\right)\} & ,\;\mathrm{if}\;\mu^{-}<x_{n}\leq 1 \end{array}\right.$$

with $\epsilon =2^{-B}$, $\mu =\epsilon \;\mathrm{floor}\{\frac{1}{\epsilon }u\}$, $\mu^{-}=\epsilon \;\mathrm{floor}\left\{\frac{1}{\epsilon }\left(1/\mu \right)\right\}$ and $\rho =\epsilon \;\mathrm{floor}\{\frac{1}{\epsilon }\;\left(\mu /\left(1-\mu \right)\right)\}$.

In [11], the authors showed the evolution of the entropy of values $H_{hist}$ with respect to the binary precision. They characterized the evolution of the TENT map as a function of binary precision in a fixed-point arithmethics. In their scheme of generation of random numbers they used two postprocessing stages, first they binarized the data by detecting the crossing by a threshold, and then these data were processed by a XOR gate. In our case we use the output of the chaotic maps without any randomization process, however their results are very interesting to take a criterion about which parameters are useful to implement. In our case, we adopted two values of *u* for two different reasons. Following [11], an interesting value is u=1.96, or its closest value within the arithmetic used. On the other hand, the value of u=2 is very attractive due to its extremely low implementation cost.

**Figure 3:**
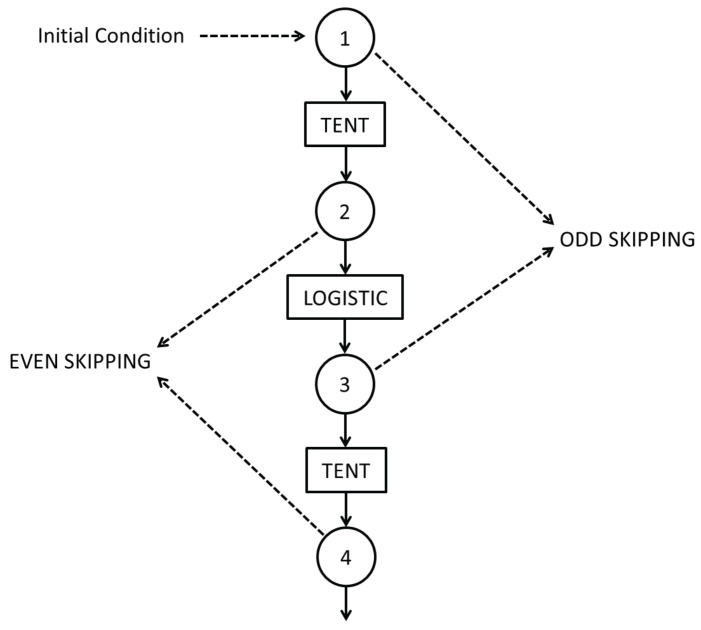
Sequential switching between Tent and Logistic maps. In the figure are also shown even and odd skipping strategies.

Switching, even and odd skipping procedures are shown in Figure 3.

SWITCH map is expressed as:

(14) $$\left\{\begin{array}{c} x_{n+1}=\left\{\begin{array}{cc} u\;x_{n} & ,\;\mathrm{if}\;0\leq x_{n}\leq 1/u\\ \frac{u}{1-u}\;\left(1-x_{n}\right) & ,\;\mathrm{if}\;1/u<x_{n}\leq 1 \end{array}\right.\\ x_{n+2}=4\;x_{n+1}\;\left(1-x_{n+1}\right) \end{array}\right.$$

with $x_{n}\in R$ and *n* an even number.

However, as in the previous cases, we work with its pseudo-chaotic counterpart that can be expressed as:

(15) $$\left\{\begin{array}{c} x_{n+1}=\left\{\begin{array}{cc} \epsilon \;\mathrm{floor}\{\frac{1}{\epsilon }\mu \;\left(x_{n}\right)\} & ,\;\mathrm{if}\;0\leq x_{n}\leq \mu^{-}\\ \epsilon \;\mathrm{floor}\{\frac{1}{\epsilon }\rho \;\left(1-x_{n}\right)\} & ,\;\mathrm{if}\;\mu^{-}<x_{n}\leq 1 \end{array}\right.\\ x_{n+2}=4\;\epsilon \;\mathrm{floor}\left\{\frac{x_{n}\left(1-x_{n}\right)}{\epsilon }\right\} \end{array}\right.$$

Skipping is a usual randomizing technique that increases the mixing quality of a single map and correspondingly increases the value of $H_{BP}$ and decreases $C_{BP}$ of the time series. Skipping does not change the values of $H_{hist}$ for ergodic maps because it are evaluated using the non causal PDF (normalized histogram of values) [23].

In the case under consideration we study even and odd skipping of the sequential switching of TENT and LOG maps:

1. Even skipping of the sequential switching of Tent and Logistic maps (EVEN).
  If $\left\{x_{n};n=1,\cdots ,\infty \right\}$ is the time series generated by Equation (15), discard all the values in odd positions and retain the values in even positions.
2. Odd skipping of the sequential switching of Tent and Logistic maps. If $\left\{x_{n};n=1,\cdots ,\infty \right\}$ is the time series generated by Equation (15), discard all the values in even positions and retain all the values in odd positions.

Even skipping may be expressed as the composition function TENT∘LOG while odd skipping may be expressed as LOG∘TENT. The evolution of period as function of precision was reported in [14] for these resulting maps. In this paper we use the same simulation algorithm and switching scheme as in [14] and we add the analysis from the statistical point of view.

### 3.1. Period as a Function of Binary Precision

Grebogi and coworkers [12] have studied how the period *T* is related with the precision. There they saw that the period *T* scales with roundoff ϵ as $T∼\epsilon^{-d/2}$ where *d* is the correlation dimension of the chaotic attractor.

Nagaraj et al. [14] studied the case of switching between two maps. They saw that the period *T* of the compound map obtained by switching between two chaotic maps is higher than the period of each map and they found that a random switching improves the results. Here we have considered sequential switching to avoid the use of another random variable, because it can include its own statistical properties in the time series.

**Figure 4:**
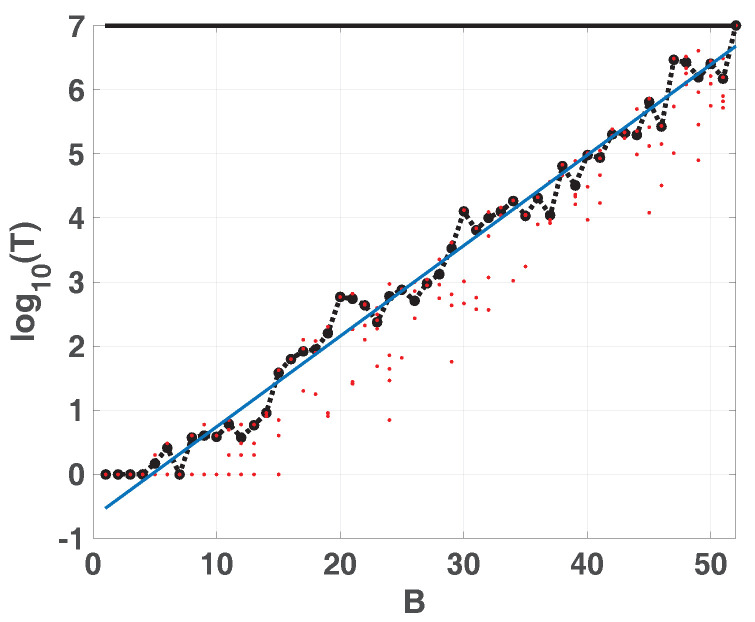
Period as function of precision in binary digits (see text).

Figure 4 shows *T* vs. *B* in semi logarithmic scale. We run the attractor of the LOG from 100 randomly chosen initial conditions. The figure show: 100 red points for each fixed-point precision (1≥B≥53) and their average (dashed black line connecting black dots). The experimental averaged points can be fitted by a straight line (in blue) expressed as $\log_{10}T=mB+b$ where *m* is the slope and *b* is the *y*-intercept.

Results for all the considered maps are summarized in Table 1. We can detect that the average period was the same using both u=2 and u=1.96 when switching strategy is used. Then we report the results for SWITCH, EVEN and ODD using TENT with u=2 to iterate.

Results are compatible for those obtained in [14]. Switching between maps increases the period *T* but skipping procedure decreases by almost half. Also, TENT with u=1.96 exhibit the longest periods.

### 3.2. Quantifiers of Simple Maps

Here we report our results for both simple maps, LOG and TENT.

#### 3.2.1. LOG

**Figure 5:**
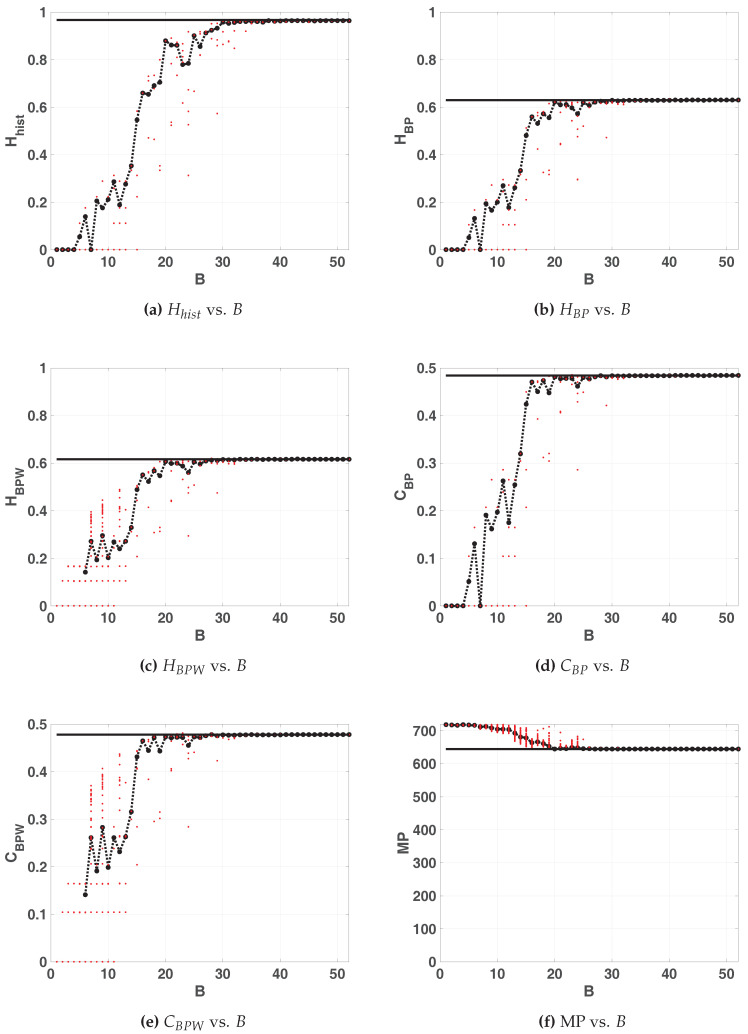
Statistical properties for LOG map as function of *B*.

Figure 5 shows the statistical properties of LOG map in floating-point and fixed-point representation. All these figures show: 100 red points for each fixed-point precision (1≥B≥53) and in black their average (dashed black line connecting black dots), 100 horizontal dashed blue lines that are the results of each run in floating-point and a black solid line their average. Note that these lines are independent of *x*-axis. In this case, all the lines of the floating-point are overlapped.

According as B grows, statistical properties vary until they stabilize. For B≥30 the value of $H_{hist}$ remains almost identical to the value for the floating-point representation whereas $H_{BP}$ and $C_{BP}$ stabilizes at B>21. Their values are: $\langle H_{hist}\rangle =0.9669$; $\langle H_{BP}\rangle =0.6269$; $\langle C_{BP}\rangle =0.4843$. Note that the stable value of missing patterns MP=645 makes the optimum $H_{BP}\leq \ln\left(75\right)/\ln\left(720\right)≃0.65$. Then, B=30 is the most convenient choice for hardware implementation because an increase in the number of fractional digits does not improve the statistical properties.

Some conclusions can be drawn regarding BP and BPW quantifiers. For B=1,2,3 and 4, the averaged BP quantifiers are almost 0 while the averaged BPW quantifiers can not be calculated (see in Figure 5c,e the missing black dashed line). This is because for those sequences were the initial condition were 0 all iterations result to be a sequence of zeros (the fixed point of the map), this is more likely to happen when using small precisions because of roundoff.

When *B* increases the initial conditions are rounded to zero less frequently, this can be seen for B>6. In this, case the generated sequences starting from a non-null value fall to zero after a short transitory very often. An interesting issue in Figure 5c,e, is that BPW quantifiers show a high dispersion unlike BP quantifiers. This is because BPW procedure takes into account the transient and discards fixed points, unlike BP procedure considers all the values of the sequence. We can see in Figure 5c,e for 1<B<10 horizontal lines of red points that do not appear in Figure 5b,d, this evidences that different initial conditions fall to the same orbits, even for adjacent precisions.

**Figure 6:**
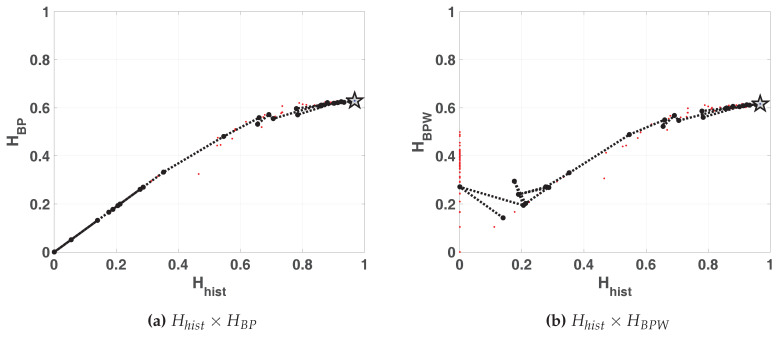
Evolution of statistical properties in double entropy plane for LOG map.

The same results are shown in double entropy planes with the precision as parameter (Figure 6a without amplitude contributions and Figure 6b with amplitude contributions). These figures show: 100 red points for each fixed-point precision (*B*) and their average in black (dashed black line connecting black dots), 100 blue dots that are the results of each run in floating-point and the black star is their average. Here, the 100 blue points and their average are overlapped.

As expected, the fixed-point architecture implementation converges to the floating-point value as *B* increases. For both planes, $H_{hist}\times H_{BP}$ and $H_{hist}\times H_{BPW}$, from B=20, $H_{hist}$ increases but $H_{BP}$ and $H_{BPW}$ remain constant. It can be seen that the distribution of values reaches high values ($\langle H_{hist}\rangle =0.9669$) but their mixing is poor ($\langle H_{BP}\rangle =0.6269$).

**Figure 7:**
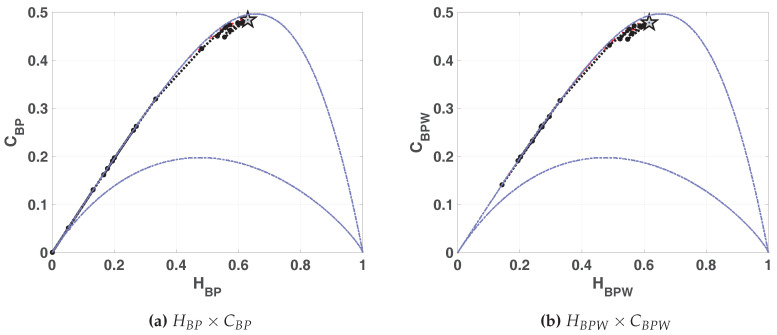
Evolution of statistical properties in causal entropy-complexity plane for LOG map.

In Figure 7a,b we show the entropy-complexity planes. Dotted grey lines are the upper and lower margins, it is expected that a chaotic system remains near the upper margin. These results characterize a chaotic behavior, in $H_{BP}\times C_{BP}$ plane we can see a low entropy and high complexity.

#### 3.2.2. TENT

The equation that represents the implementation for u=2 is:

(16) $$x_{n+1}=\left\{\begin{array}{cc} 2\;x_{n} & ,\;\mathrm{if}\;0\leq x_{n}\leq 0.5\\ \epsilon \;\mathrm{floor}\{\frac{1}{\epsilon }2\;\left(1-x_{n}\right)\} & ,\;\mathrm{if}\;0.5<x_{n}\leq 1 \end{array}\right.$$

Rounding is necessary only on the second multiplication because it is equivalent to a shift-to-left.

When this map is implemented with u=2 in any computer using any numerical representation system (even floating-point!) truncation errors rapidly increase and make the unstable fixed point in $x^{*}=0$ to become stable. The sequences within the attractor domain of this fixed point will have a short transitory of length between 0 and *B* followed by an infinite number of 0’s [50,51]. This issue is easily explained in [52], the problem appears because all iterations have a left-shift operation that carries the 0’s from the right side of the number to the most significant positions. As expected, the quantifiers $H_{hist}$, $H_{BP}$ and $C_{BP}$ are equal to zero for all precisions. In the case of $H_{BPW}$ and $C_{BPW}$ quantifiers are different to zero because BPW procedure discards the elements once the fixed point is reached. $H_{BPW}$, $C_{BPW}$ and MP present high dispersions related to the short length of serie’s transient. These transients have a maximum length of *B* elements (iterations) for fixed-point arithmetic and 53 for floating-point (IEEE754 double precision).

**Figure 8:**
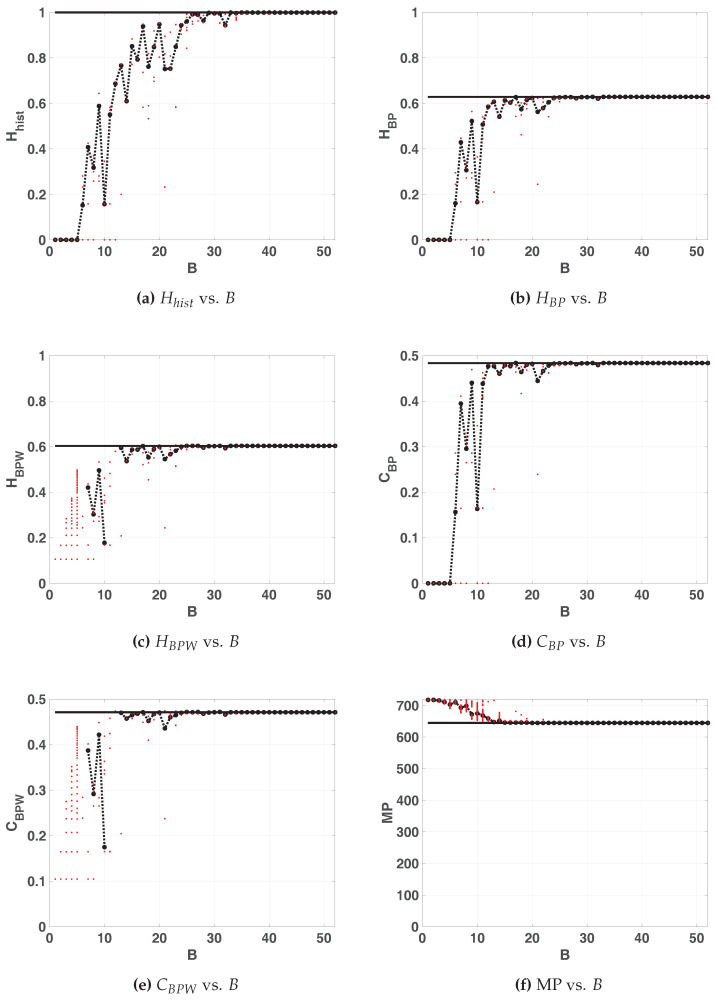
Statistical properties of TENT map with paramater u=1.96.

Figure 8 shows the quantifiers for floating- and fixed-point numerical representations for u=1.96. The results are similar to those of the LOG map, in that the averaged value of the quantifiers tends not monotonously to the floating point value and stabilizes from a certain value of *B*. However, more bits of precision were necessary to achieve this, on the other hand it can be seen that the value of $H_{hist}$ improves and that of $H_{BP}$ remains similar to those of the LOG map. Using $H_{BPW}$ and $C_{BPW}$ we detect that below the 13 precision bits some initial conditions converge to fixed points or diverge, so it is not possible to use this map with B<13.

**Figure 9:**
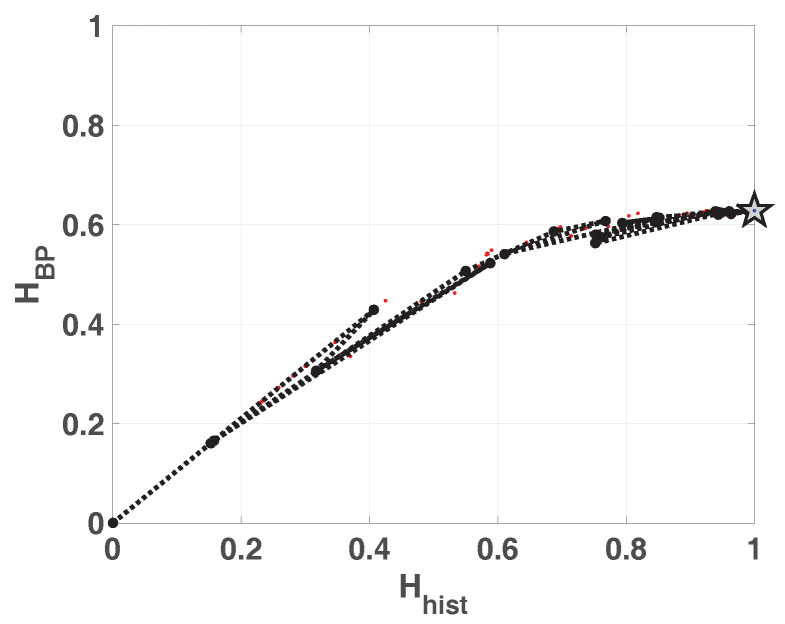
Evolution of statistical properties in double entropy plane for TENT map with u=1.96.

**Figure 10:**
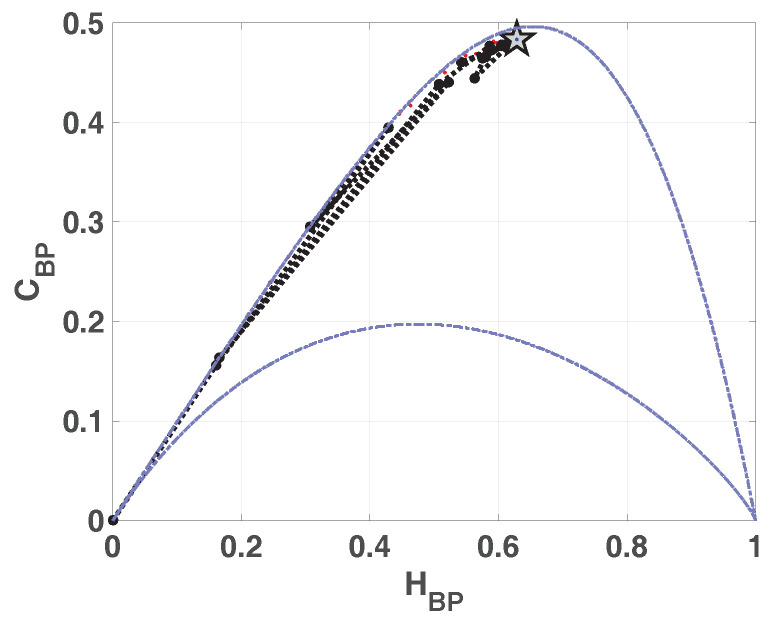
Evolution of statistical properties in causal entropy-complexity plane for TENT map with u=1.96.

The positions on the double entropy (Figure 9) and entropy complexity (Figure 10) planes are marginally better than those on the LOG map and are characteristic of a pseudo-chaotic system.

### 3.3. Quantifiers of Combined Maps

Here we report our results for the three combinations of the combined maps, SWITCH, EVEN and ODD.

#### 3.3.1. SWITCH

Between the two parameters analyzed for the TENT map, we found that mixing and stochasticity converge to the same values either for u=1.96 or for u=2. So, we chose to use u=2 given its simplicity to be used both in software simulations and in hardware implementation.

**Figure 11:**
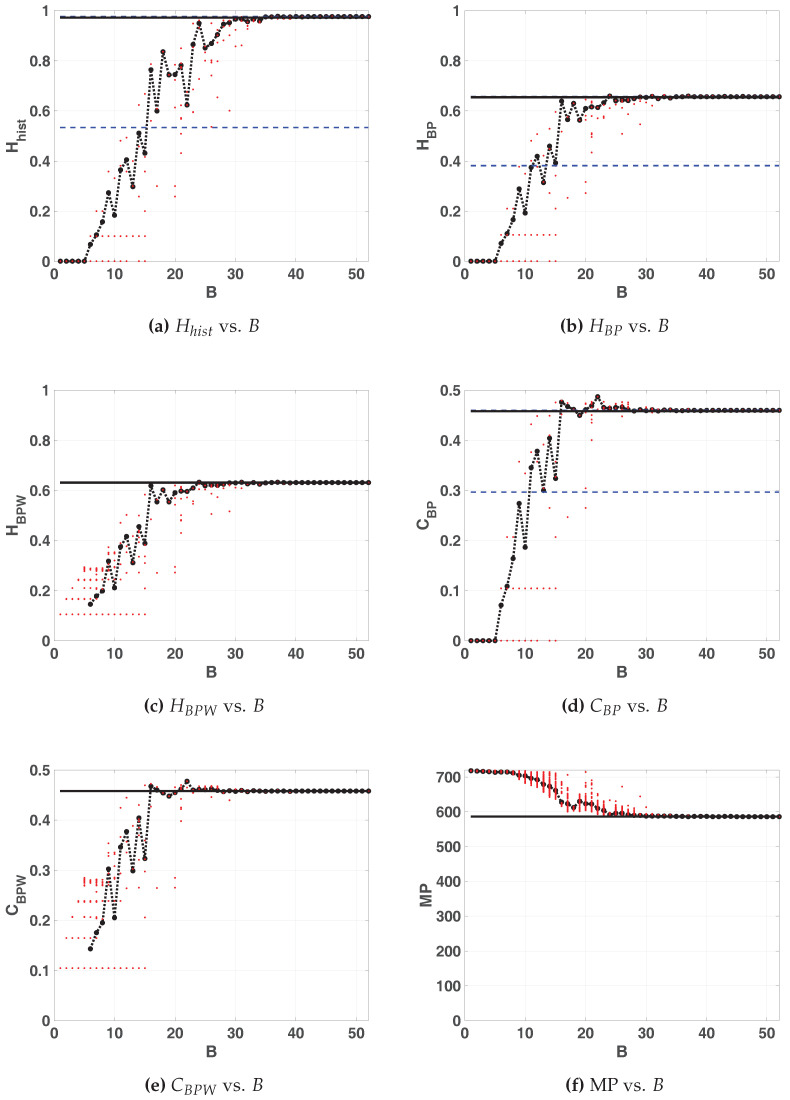
Statistical properties of SWITCH map.

Results with sequential switching are shown in Figure 11. The calculated entropy value for floating-point implementation is $\langle H_{hist}\rangle =0.9722$, this value is slightly higher than the one obtained for the LOG map. For fixed-point arithmetic this value is reached in B=24, but it stabilizes from B=28. Regarding the ordering patterns the number of MP decreases to 586, this value is lower than the one obtained for LOG map. It means the entropy $H_{BP}$ may increase up to ln(134)/ln(720)≃0.74. BP and BPW quantifiers reach their maximum of $\langle H_{BP}\rangle =0.6546$ and $\langle H_{BPW}\rangle =0.6313$ at B=16, but they stabilize from B=24. Complexities are lower than for LOG, $\langle C_{BP}\rangle =0.4580$ and $\langle C_{BPW}\rangle =0.4578$, these values are reached for B≥15 but they stabilize for B≥23. Compared with LOG, statistical properties are better with less amount of bits, for B≥24 this map reaches optimal characteristics in the sense of random source.

Furthermore, we encountered one initial condition in floating-point with an anomalous behavior. Figure 11a,b,d show an horizontal blue dashed line that is far from the average value, unlike this is not detected by quantifiers based on BPW procedure in Figure 11c,e. Nevertheless comparing both procedures (BP and BPW) we were able to detect a falling to fixed point after a long transitory, the BPW procedure discards the constant values (corresponding with a fixed point) and works only over the transitory values.

**Figure 12:**
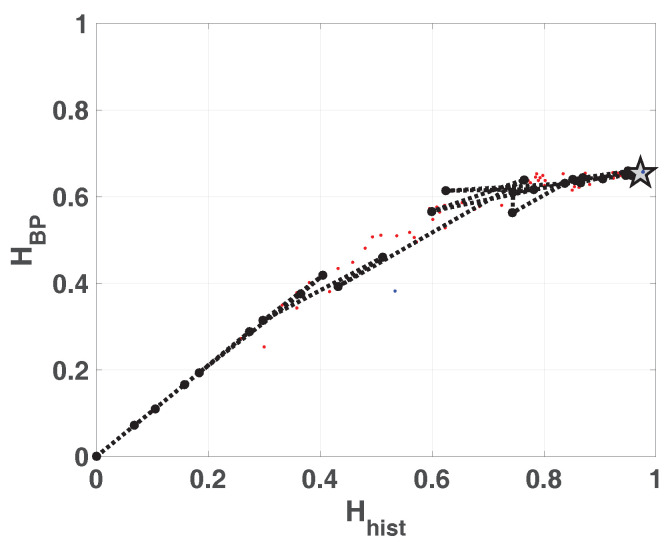
Evolution of statistical properties in double entropy plane for SWITCH map $H_{hist}\times H_{BP}$.

Double entropy plane $H_{hist}\times H_{BP}$ is showed in Figure 12. The reached point in this plane for SWITCH map is similar to that reached for LOG map, and it is denoted by a star in the figure. The mixing is slight better in this case.

**Figure 13:**
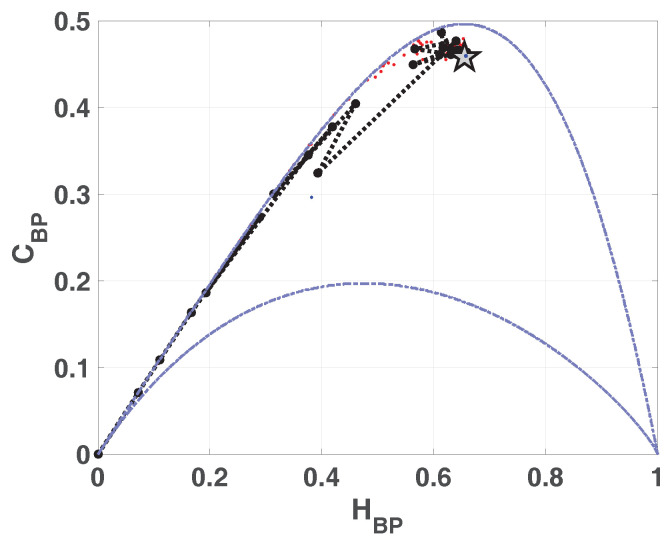
Evolution of statistical properties in causal entropy-complexity plane for SWITCH map $H_{BP}\times C_{BP}$.

Entropy-complexity plane $H_{BP}\times C_{BP}$ is showed in Figure 13. If we compare with the same plane in the case of LOG (Figure 7a), $C_{BP}$ is lower for SWITCH, this fact shows a more random behavior.

#### 3.3.2. EVEN and ODD

**Figure 14:**
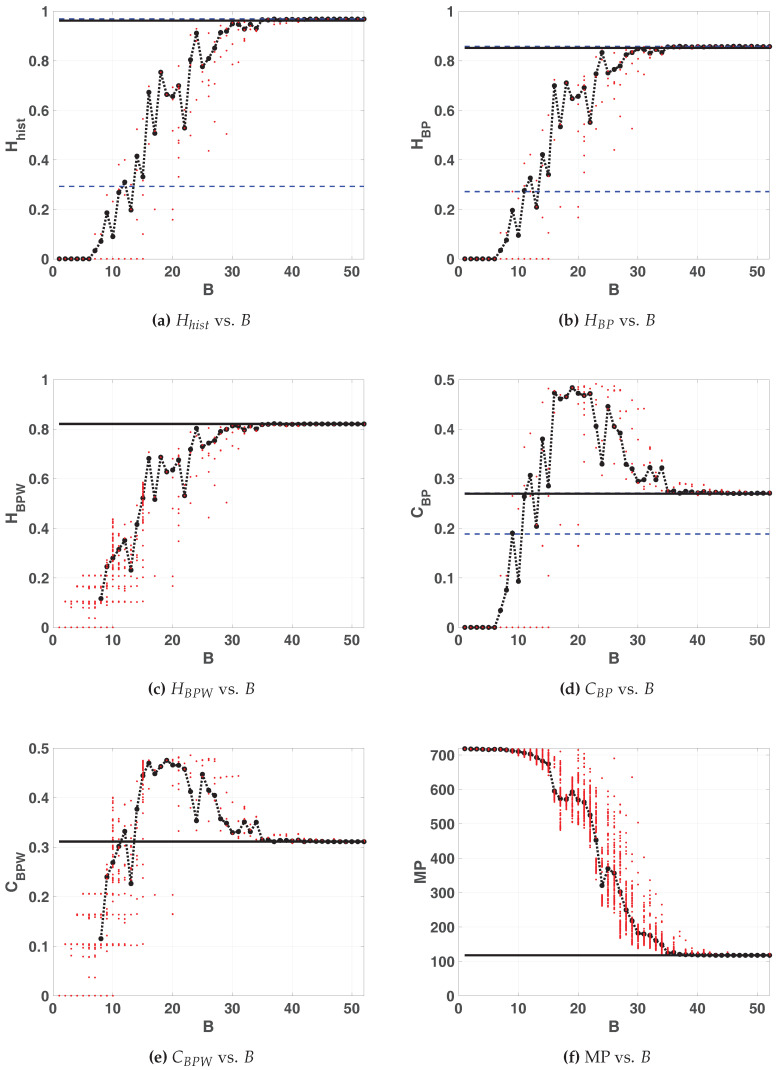
Statistical properties of EVEN map.

**Figure 15:**
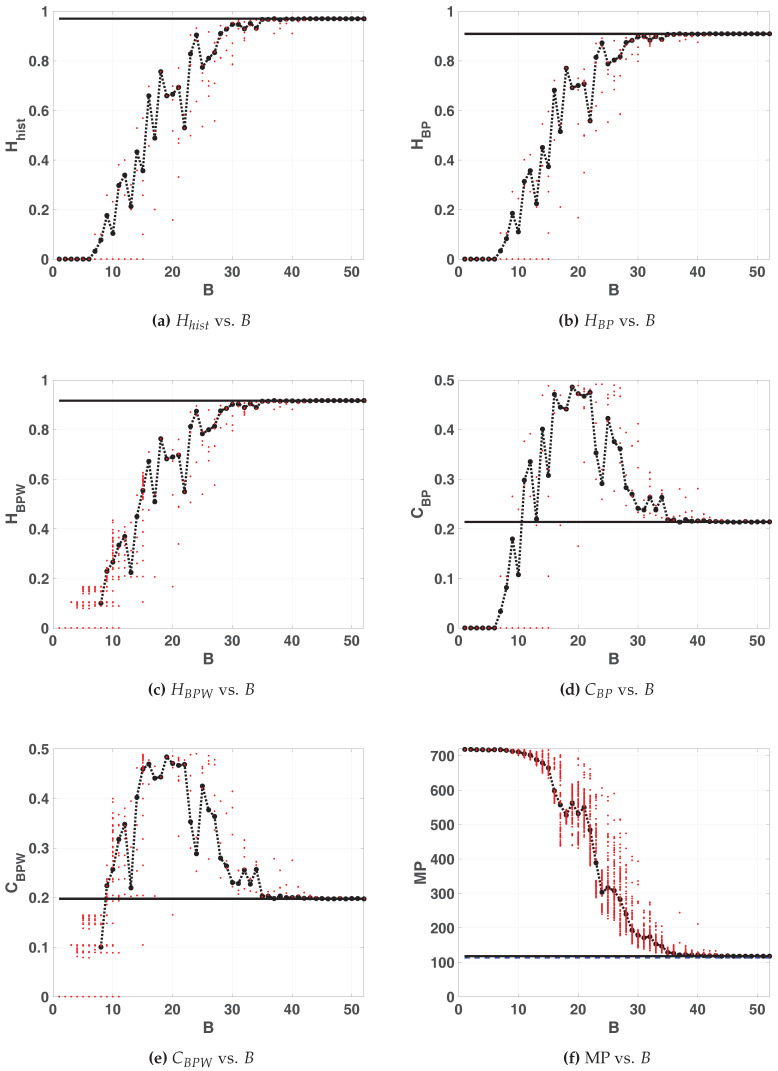
Statistical properties of ODD map.

In Figure 14a and Figure 15a we can see that quantifiers related to the normalized histogram of values slightly degrades with the skipping procedure. For example $\langle H_{hist}\rangle$ reduces from 0.9722 without skipping to 0.9459 for EVEN and 0.9706 for ODD. This difference between EVEN and ODD in floating point is because a high dispersion was obtained for $H_{hist}$, $H_{BP}$ and $C_{BP}$ but not for $H_{BPW}$ or $C_{BPW}$.

Figure 14b–f and Figure 15b–f show the results of BP and BPW quantifiers for EVEN and ODD respectively. Higher accuracy is required to achieve lower complexity than without using skipping. From the MP point of view a great improvement is obtained using any of the skipping strategies but ODD is slightly better than EVEN. Missing patterns are reduced to MP=118 for EVEN and ODD, increasing the maximum allowed Bandt & Pompe entropy that reaches the mean value $\langle H_{BP}\rangle =0.8381$ for EVEN, and $\langle H_{BP}\rangle =0.9094$. The complexity is reduced to $\langle C_{BP}\rangle =0.224$ for EVEN and $\langle C_{BP}\rangle =0.282$ for ODD. The minimum number of bits to converge to this value is B>40 for both EVEN and ODD maps.

**Figure 16:**
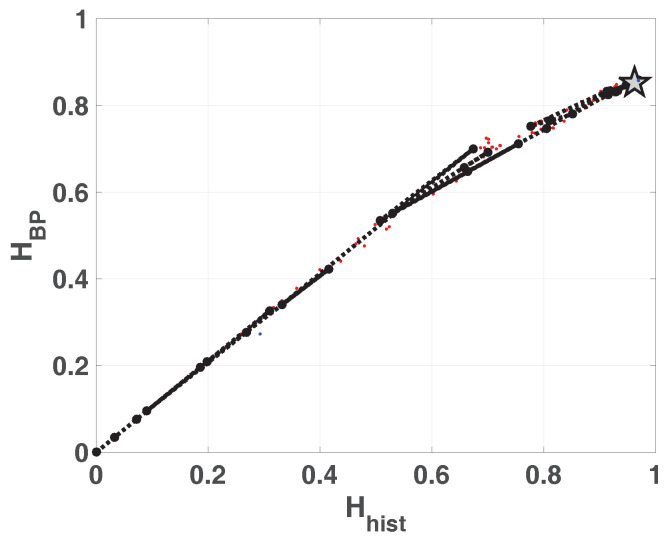
Evolution of statistical properties in double entropy plane for EVEN map $H_{hist}\times H_{BP}$.

**Figure 17:**
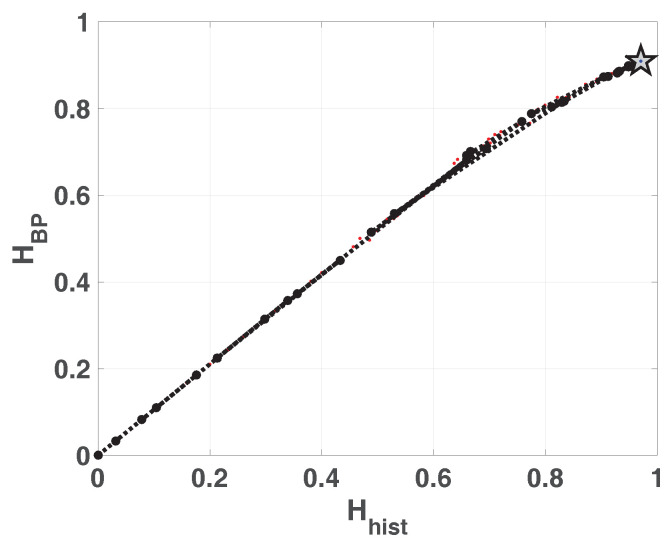
Evolution of statistical properties in double entropy plane for ODD map $H_{hist}\times H_{BP}$.

The enhancement showed in Figure 14 and Figure 15 is reflected in the position of asymptotic point in the planes Figure 16 and Figure 17. In both cases this position is closest to the ideal point $\left(H_{hist},H_{BP}\right)=\left(1,1\right)$, because the resulting vectors present better mixing.

**Figure 18:**
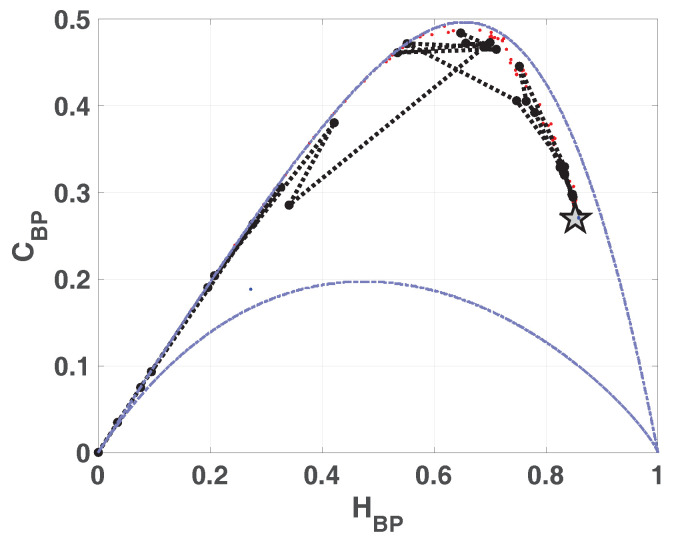
Evolution of statistical properties in entropy-complexity plane for EVEN map $H_{BP}\times C_{BP}$.

**Figure 19:**
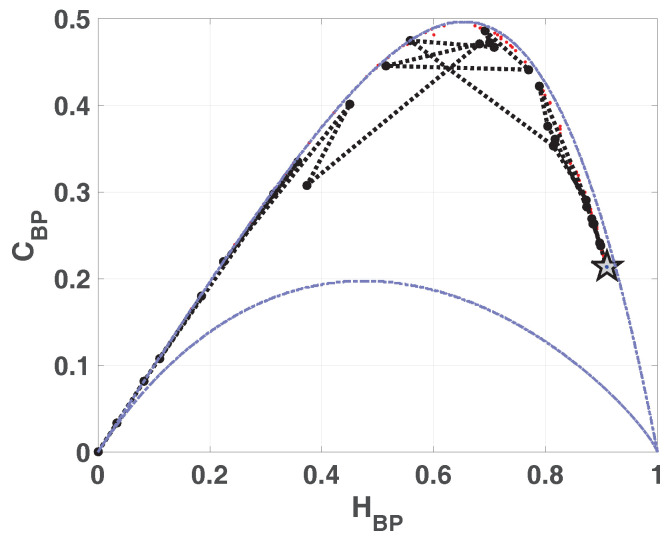
Evolution of statistical properties in entropy-complexity plane for ODD map $H_{BP}\times C_{BP}$.

Compatible results are shown in Figure 18 and Figure 19, the position of asymptotic point is closest to the ideal point $\left(H_{hist},H_{BP}\right)=\left(1,0\right)$. This result reflects that mixing is better because the complexity of resulting system is lower. This plane detects that the vector generated by ODD skipping is more mixed than EVEN.

## 4. Conclusions

In this paper we explored the statistical degradation of simple, switched and skipped chaotic maps due to the inherent error of a based-2 systems. We evaluated mixing and amplitude distributions from a statistical point of view.

Our work complements previous results given in [14], where period lengths were investigated. In that sense, our results were compatible with these. We can see that the switching between two maps increases the dependence of period as function of precision, nevertheless the standard procedure of skipping reduce the period length in almost a half.

All statistics of the maps represented in fixed-point produces a non-monotonous evolution toward the floating-point results. This result is relevant because it shows that increasing the precision is not always recommended.

Our results show that SWITCH has a marginal improvement in the mixing with respect to LOG and TENT. However the greatest improvement comes when skipping is applied, we can see that BP and BPW entropies grow and BP and BPW complexities decrease, for the same numerical representation. This result is relevant because evidences that a long period is not synonymous of good statistics, switched maps EVEN and ODD have half period lengths but their mixing is better and their amplitude distributions remain almost equal. As counterpart, more precision is needed to reach the better asymptotes that offers the switching method.

It was especially interesting to note that the TENT maps with u=2 (which produces outputs that quickly converge to zero) and u=1.96 (with statistical properties better than LOG), produce outputs with the same results when they are included in the switched scheme.

## Figures and Tables

**Table 1:** Period *T* as a function of *B* for the maps considered. SWITCH, EVEN and ODD were calculated with u=2 (see text).

Map	*m*	*b*
TENT u=2	0	0
TENT u=1.96	0.1487	−0.01177
LOG	0.139	−0.6188
SWITCH	0.1462	−0.5115
EVEN	0.1447	−0.7783
ODD	0.1444	−0.7683
